# Supplementary material for: Increased anxiety and decreased sociability induced by paternal deprivation involve the PVN-PrL OTergic pathway
Source: eLife. 2019 May 14;8:e44026. doi: 10.7554/eLife.44026 (PMC6516825; doi:10.7554/eLife.44026)
Supplement: Figure 1—source data 1. [file elife-44026-fig1-data1.docx]

**Source Data for Figure 1A, B, E, F**

| **Sex** | **Treatment** | **Time in the central area (%)** | **Total distance (cm)** |
| --- | --- | --- | --- |
| **Male** | **PC** | 14.03 | 1690.61 |
|  |  | 11.74 | 3423.26 |
|  |  | 27.87 | 3320.45 |
|  |  | 9.75 | 1564.05 |
|  |  | 31.16 | 2315.13 |
|  |  | 20.33 | 2090.39 |
|  |  | 18.77 | 4585.96 |
|  | **PD** | 2.58 | 1333.15 |
|  |  | 10.43 | 2759.54 |
|  |  | 3.56 | 2746.8 |
|  |  | 6.21 | 2387.18 |
|  |  | 5.84 | 2618.12 |
|  |  | 4.96 | 1874.55 |
|  |  | 6.92 | 2581.31 |
|  | **PC vs.PD** | **P < 0.01** | **P = 0.43** |
| **Female** | **PC** | 38.18 | 2125.47 |
|  |  | 29.08 | 2184.48 |
|  |  | 25.08 | 1371.14 |
|  |  | 10.76 | 2355.94 |
|  |  | 10.71 | 2385.8 |
|  |  | 13.4 | 4096.16 |
|  |  | 12.23 | 1701.96 |
|  | **PD** | 11.87 | 3073.84 |
|  |  | 3.27 | 1777.2 |
|  |  | 1.14 | 2280.83 |
|  |  | 7.31 | 3850.6 |
|  |  | 4.61 | 1728.94 |
|  |  | 4.02 | 2474.18 |
|  |  | 8.91 | 2016.76 |
|  | **PC vs.PD** | **P < 0.05** | **P= 0.75** |

**Source Data for Figure 1G, H**

| **Sex** | **Treatment** | **Time in light (%)** |
| --- | --- | --- |
| **Male** | **PC** | 93.53 |
|  |  | 62.91 |
|  |  | 84.29 |
|  |  | 52.11 |
|  |  | 72.94 |
|  |  | 57.19 |
|  |  | 65.00 |
|  | **PD** | 30.72 |
|  |  | 28.59 |
|  |  | 46.21 |
|  |  | 8.36 |
|  |  | 51.04 |
|  |  | 78.73 |
|  |  | 55.66 |
|  | **PC vs.PD** | **P < 0.05** |
| **Female** | **PC** | 90.14 |
|  |  | 53.52 |
|  |  | 67.69 |
|  |  | 44.48 |
|  |  | 63.23 |
|  |  | 90.27 |
|  |  | 66.30 |
|  | **PD** | 40.14 |
|  |  | 49.68 |
|  |  | 21.42 |
|  |  | 48.66 |
|  |  | 40.38 |
|  |  | 31.33 |
|  |  | 37.75 |
|  | **PC vs.PD** | **P < 0.01** |

**Source Data for Figure 1K, L**

| **Sex** | **Treatment** | **Object (%)** | **Social (%)** | **Object vs. Social** |
| --- | --- | --- | --- | --- |
| **Male** | **PC** | 38.53 | 65.09 | **P < 0.025** |
|  |  | 27.64 | 58.85 |  |
|  |  | 29.77 | 66.23 |  |
|  |  | 28.78 | 93.92 |  |
|  |  | 62.33 | 95.15 |  |
|  |  | 21.74 | 89.11 |  |
|  |  | 39.8 | 59.27 |  |
|  | **PD** | 41.41 | 52.12 | **P = 0.053** |
|  |  | 19.36 | 69.04 |  |
|  |  | 26.12 | 15.87 |  |
|  |  | 33.54 | 43.49 |  |
|  |  | 35.31 | 38.59 |  |
|  |  | 35.49 | 67.16 |  |
|  |  | 38.52 | 54.38 |  |
|  | **PC vs. PD** | **n.s.** | **P < 0.01** |  |
| **Female** | **PC** | 33.21 | 40.56 | **P < 0.025** |
|  |  | 19.34 | 63.44 |  |
|  |  | 18.56 | 43.58 |  |
|  |  | 33.76 | 89.04 |  |
|  |  | 56.25 | 57.85 |  |
|  |  | 33.36 | 65.79 |  |
|  |  | 21.86 | 56.66 |  |
|  | **PD** | 45.88 | 59 | **P = 0.141** |
|  |  | 27.49 | 21.16 |  |
|  |  | 27.98 | 29.83 |  |
|  |  | 25.69 | 62.79 |  |
|  |  | 14.47 | 6.54 |  |
|  |  | 16.11 | 62.16 |  |
|  |  | 37.1 | 46.99 |  |
|  | **PC vs. PD** | **n.s.** | **n.s.** |  |
