# Supplementary material for: Increased anxiety and decreased sociability induced by paternal deprivation involve the PVN-PrL OTergic pathway
Source: eLife. 2019 May 14;8:e44026. doi: 10.7554/eLife.44026 (PMC6516825; doi:10.7554/eLife.44026)
Supplement: Figure 2—source data 1. [file elife-44026-fig2-data1.docx]

**Source Data for Figure 2E, F**

| **Sex** | **Treatment** | **First**  **section**  **(fos/OT)** | | **Second**  **section**  **(fos/OT)** | | **Third**  **Section**  **(fos/OT)** | | **Fourth**  **Section**  **(fos/OT)** | | **Fifth**  **Section**  **(fos/OT)** | | **Sixth**  **Section**  **(fos/OT)** | | **Mean**  **(%)** | | **Object vs. Social** |
| --- | --- | --- | --- | --- | --- | --- | --- | --- | --- | --- | --- | --- | --- | --- | --- | --- |
|  |  | **Object** | **Social** | **Object** | **Social** | **Object** | **Social** | **Object** | **Social** | **Object** | **Social** | **Object** | **Social** | **Object** | **Social** |  |
| **Male** | **PC** | 2/27 | 11/65 | 2/50 | 10/66 | 3/58 | 8/39 | 4/35 | 11/46 | 3/42 | 11/66 | 5/48 | 6/49 | 7.31 | 17.22 | P < 0.025 |
|  |  | 1/71 | 5/40 | 4/57 | 7/50 | 1/44 | 6/49 | 2/41 | 7/30 | 2/39 | 1/32 | 3/54 | 2/38 | 4.25 | 11.72 |  |
|  |  | 1/67 | 4/52 | 0/51 | 6/59 | 1/42 | 3/32 | 2/27 | 4/46 | 0/34 | 4/54 | 2/38 | 2/36 | 2.32 | 8.24 |  |
|  |  | 2/67 | 5/63 | 5/54 | 7/78 | 1/41 | 9/49 | 3/36 | 7/37 | 2/52 | 6/46 | 1/69 | 6/31 | 4.39 | 13.16 |  |
|  |  | 3/82 | 5/46 | 2/65 | 5/57 | 1/43 | 4/35 | 1/52 | 6/43 | 2/32 | 5/49 | 3/51 | 3/42 | 3.28 | 10.29 |  |
|  | **PD** | 2/83 | 6/36 | 4/55 | 6/54 | 3/43 | 1/47 | 2/27 | 2/33 | 4/36 | 4/35 | 2/37 | 2/30 | 6.05 | 8.94 | P=0. 877 |
|  |  | 4/45 | 4/26 | 7/74 | 11/64 | 6/53 | 9/65 | 3/20 | 6/33 | 3/24 | 6/42 | 4/31 | 4/35 | 10.93 | 15.09 |  |
|  |  | 3/48 | 1/51 | 7/55 | 1/96 | 9/47 | 2/58 | 5/30 | 1/52 | 1/28 | 0/30 | 1/24 | 4/31 | 11.21 | 2.83 |  |
|  |  | 1/34 | 1/49 | 2/65 | 2/87 | 3/76 | 2/62 | 4/44 | 1/57 | 3/43 | 1/26 | 4/25 | 2/25 | 5.92 | 2.94 |  |
|  |  | 3/45 | 3/35 | 5/81 | 10/72 | 3/43 | 8/57 | 3/36 | 5/37 | 1/26 | 6/39 | 4/34 | 5/38 | 7.17 | 13.31 |  |
|  | **PC vs. PD** |  | | | | | | | | | | | | **Object** : P = 0. 109  **Social** : P = 0. 152 | |  |
| **Female** | **PC** | 4/75 | 22/64 | 11/55 | 21/78 | 4/52 | 14/67 | 5/52 | 7/36 | 3/35 | 4/35 | 6/105 | 9/44 | 8.82 | 23.77 | P < 0.025 |
|  |  | 4/59 | 9/64 | 5/79 | 5/70 | 7/39 | 8/49 | 15/110 | 5/48 | 14/85 | 7/52 | 4/50 | 3/18 | 11.61 | 12.29 |  |
|  |  | 3/35 | 18/69 | 5/86 | 12/51 | 6/58 | 4/34 | 1/62 | 3/25 | 3/35 | 3/27 | 2/45 | 5/35 | 6.23 | 18.67 |  |
|  |  | 5/82 | 6/54 | 6/48 | 12/87 | 7/63 | 15/98 | 2/54 | 18/67 | 10/92 | 5/72 | 3/63 | 6/35 | 8.21 | 15.01 |  |
|  |  | 5/58 | 7/67 | 4/92 | 13/104 | 8/83 | 9/78 | 3/84 | 8/54 | 4/37 | 6/36 | 2/45 | 6/42 | 6.52 | 12.86 |  |
|  | **PD** | 3/34 | 3/31 | 7/47 | 6/40 | 2/61 | 3/44 | 5/50 | 7/45 | 2/30 | 4/53 | 1/35 | 0/29 | 7.78 | 9.50 | P=0. 854 |
|  |  | 1/38 | 2/58 | 0/32 | 4/48 | 5/40 | 6/32 | 1/11 | 2/18 | 2/23 | 1/40 | 2/36 | 2/42 | 6.11 | 7.14 |  |
|  |  | 0/20 | 2/50 | 4/24 | 3/40 | 4/46 | 1/39 | 4/37 | 0/21 | 6/41 | 2/34 | 4/52 | 1/23 | 10.00 | 4.35 |  |
|  |  | 4/42 | 3/42 | 6/51 | 2/62 | 4/54 | 2/34 | 2/59 | 1/45 | 4/34 | 3/32 | 2/42 | 3/28 | 7.80 | 5.76 |  |
|  |  | 0/35 | 2/36 | 2/42 | 4/44 | 4/57 | 5/53 | 2/63 | 6/43 | 3/32 | 5/67 | 2/27 | 1/35 | 5.08 | 8.27 |  |
|  | **PC vs. PD** |  | | | | | | | | | | | | **Object** : P = 0. 628  **Social** : P < 0. 025 | |  |
