# Supplementary material for: Increased anxiety and decreased sociability induced by paternal deprivation involve the PVN-PrL OTergic pathway
Source: eLife. 2019 May 14;8:e44026. doi: 10.7554/eLife.44026 (PMC6516825; doi:10.7554/eLife.44026)
Supplement: Figure 3—source data 1. [file elife-44026-fig3-data1.docx]

**Source Data for Figure 3D, E**

| **Sex** | **Treatment** | **Target** | **First**  **section**  **(#)** | | **Second**  **section**  **(#)** | | **Third**  **section**  **(#)** | | **Fourth**  **section**  **(#)** | | **Fifth**  **section**  **(#)** | | **Sixth**  **section**  **(#)** | | **Total** | **Mean**  **(#/mm2)** | **Object** vs. **Social** |
| --- | --- | --- | --- | --- | --- | --- | --- | --- | --- | --- | --- | --- | --- | --- | --- | --- | --- |
|  |  |  | Right | Left | Right | Left | Right | Left | Right | Left | Right | Left | Right | Left |  |  |  |
| **Male** | **PC** | **Object** | 57 | 54 | 57 | 36 | 66 | 62 | 43 | 46 | 88 | 50 | 70 | 31 | 660 | 179.04 | P < 0.01 |
|  |  | **Social** | 236 | 102 | 157 | 89 | 135 | 59 | 46 | 58 | 47 | 8 | 50 | 48 | 1035 | 280.76 |  |
|  |  | **Object** | 30 | 28 | 57 | 62 | 52 | 38 | 42 | 28 | 43 | 43 | 28 | 37 | 488 | 132.38 |  |
|  |  | **Social** | 73 | 67 | 136 | 168 | 92 | 48 | 65 | 76 | 28 | 6 | 64 | 25 | 848 | 230.03 |  |
|  |  | **Object** | 29 | 40 | 61 | 53 | 63 | 78 | 45 | 53 | 36 | 40 | 10 | 6 | 514 | 139.43 |  |
|  |  | **Social** | 84 | 82 | 67 | 58 | 49 | 23 | 57 | 72 | 86 | 68 | 74 | 58 | 778 | 211.05 |  |
|  |  | **Object** | 43 | 47 | 51 | 28 | 36 | 42 | 35 | 28 | 57 | 41 | 55 | 24 | 487 | 132.11 |  |
|  |  | **Social** | 89 | 74 | 67 | 84 | 93 | 119 | 172 | 65 | 54 | 19 | 82 | 67 | 985 | 267.20 |  |
|  |  | **Object** | 27 | 21 | 42 | 43 | 61 | 21 | 34 | 23 | 35 | 31 | 34 | 18 | 390 | 105.79 |  |
|  |  | **Social** | 72 | 64 | 81 | 62 | 56 | 34 | 73 | 46 | 72 | 61 | 52 | 36 | 709 | 192.33 |  |
|  | **PD** | **Object** | 18 | 19 | 8 | 13 | 2 | 14 | 1 | 5 | 12 | 12 | 8 | 10 | 122 | 33.09 | P = 0. 916 |
|  |  | **Social** | 4 | 3 | 10 | 2 | 4 | 6 | 1 | 3 | 9 | 7 | 10 | 7 | 66 | 17.9 |  |
|  |  | **Object** | 4 | 3 | 3 | 11 | 12 | 12 | 3 | 17 | 7 | 18 | 6 | 5 | 101 | 27.4 |  |
|  |  | **Social** | 141 | 89 | 28 | 22 | 31 | 26 | 36 | 23 | 36 | 6 | 57 | 22 | 517 | 140.25 |  |
|  |  | **Object** | 42 | 40 | 18 | 36 | 125 | 25 | 55 | 33 | 42 | 62 | 47 | 67 | 592 | 160.59 |  |
|  |  | **Social** | 42 | 43 | 56 | 42 | 7 | 6 | 16 | 22 | 33 | 18 | 29 | 16 | 330 | 89.52 |  |
|  |  | **Object** | 23 | 25 | 9 | 24 | 16 | 3 | 26 | 7 | 24 | 31 | 36 | 31 | 255 | 69.17 |  |
|  |  | **Social** | 35 | 52 | 45 | 34 | 19 | 35 | 34 | 21 | 19 | 12 | 7 | 9 | 322 | 71.89 |  |
|  |  | **Object** | 32 | 23 | 26 | 38 | 76 | 47 | 42 | 44 | 32 | 63 | 27 | 34 | 484 | 131.29 |  |
|  |  | **Social** | 24 | 31 | 6 | 7 | 4 | 5 | 23 | 32 | 16 | 29 | 52 | 36 | 265 | 87.35 |  |
|  | **PC vs. PD** |  | | | | | | | | | | | | | | **Object** : P = 0. 069  **Social** : P < 0.01 |  |
| **Female** | **PC** | **Object** | 32 | 34 | 47 | 33 | 25 | 33 | 16 | 9 | 39 | 45 | 49 | 37 | 399 | 108.24 | P < 0.01 |
|  |  | **Social** | 72 | 63 | 82 | 40 | 71 | 44 | 60 | 50 | 46 | 33 | 66 | 54 | 681 | 184.73 |  |
|  |  | **Object** | 24 | 26 | 46 | 36 | 40 | 42 | 33 | 27 | 37 | 21 | 32 | 39 | 403 | 109.32 |  |
|  |  | **Social** | 245 | 217 | 130 | 124 | 152 | 135 | 156 | 116 | 86 | 123 | 67 | 60 | 1611 | 437.01 |  |
|  |  | **Object** | 45 | 62 | 65 | 54 | 30 | 40 | 63 | 55 | 27 | 41 | 10 | 4 | 496 | 134.55 |  |
|  |  | **Social** | 88 | 92 | 60 | 45 | 52 | 82 | 103 | 60 | 65 | 61 | 58 | 117 | 883 | 239.53 |  |
|  |  | **Object** | 41 | 36 | 42 | 27 | 31 | 34 | 19 | 7 | 28 | 61 | 37 | 62 | 425 | 115.29 |  |
|  |  | **Social** | 189 | 241 | 114 | 167 | 108 | 153 | 132 | 174 | 76 | 65 | 84 | 63 | 1566 | 424.8 |  |
|  |  | **Object** | 39 | 46 | 62 | 16 | 22 | 24 | 42 | 57 | 36 | 39 | 5 | 7 | 395 | 107.15 |  |
|  |  | **Social** | 84 | 61 | 54 | 69 | 92 | 73 | 51 | 73 | 37 | 46 | 75 | 64 | 779 | 211.32 |  |
|  | **PD** | **Object** | 3 | 3 | 4 | 6 | 11 | 6 | 10 | 3 | 15 | 5 | 21 | 14 | 101 | 27.4 | P = 0. 665 |
|  |  | **Social** | 6 | 5 | 6 | 12 | 13 | 12 | 23 | 21 | 34 | 13 | 36 | 6 | 187 | 50.73 |  |
|  |  | **Object** | 43 | 30 | 55 | 45 | 51 | 9 | 10 | 15 | 45 | 44 | 50 | 28 | 397 | 107.69 |  |
|  |  | **Social** | 67 | 58 | 39 | 43 | 46 | 15 | 73 | 62 | 67 | 63 | 69 | 37 | 639 | 173.34 |  |
|  |  | **Object** | 11 | 14 | 40 | 27 | 31 | 25 | 20 | 15 | 62 | 22 | 58 | 22 | 347 | 94.13 |  |
|  |  | **Social** | 48 | 56 | 17 | 5 | 6 | 8 | 8 | 15 | 16 | 17 | 10 | 18 | 224 | 60.76 |  |
|  |  | **Object** | 24 | 16 | 34 | 24 | 64 | 24 | 18 | 13 | 25 | 63 | 46 | 35 | 386 | 104.71 |  |
|  |  | **Social** | 54 | 62 | 34 | 57 | 32 | 21 | 67 | 35 | 37 | 72 | 52 | 34 | 557 | 151.1 |  |
|  |  | **Object** | 34 | 23 | 42 | 32 | 21 | 16 | 15 | 7 | 36 | 25 | 26 | 19 | 296 | 80.3 |  |
|  |  | **Social** | 12 | 16 | 22 | 21 | 16 | 11 | 23 | 19 | 35 | 48 | 27 | 26 | 276 | 74.87 |  |
|  | **PC vs. PD** |  | | | | | | | | | | | | | | **Object** : P = 0.474  **Social** : P < 0.01 |  |

**Source Data for Figure 3F, G, H, I**

| NAc | Sex | Treatment | Target | First  section  (#) | Second  section  (#) | Third  section  (#) | Fourth  section  (#) | Fifth  section  (#) | Sixth  section  (#) | Total | Mean  (#/mm^2^) | **Object** vs. **Social** |
| --- | --- | --- | --- | --- | --- | --- | --- | --- | --- | --- | --- | --- |
| **Shell** | **Male** | **PC** | **Object** | 62 | 75 | 57 | 76 | 54 | 52 | 376 | 296.72 | P = 0.353 |
|  |  |  | **Social** | 49 | 80 | 89 | 40 | 72 | 72 | 402 | 317.23 |  |
|  |  |  | **Object** | 45 | 16 | 17 | 54 | 48 | 44 | 224 | 176.77 |  |
|  |  |  | **Social** | 33 | 35 | 39 | 16 | 58 | 55 | 236 | 186.24 |  |
|  |  |  | **Object** | 29 | 28 | 34 | 42 | 38 | 23 | 194 | 153.09 |  |
|  |  |  | **Social** | 48 | 19 | 27 | 33 | 46 | 25 | 198 | 156.25 |  |
|  |  |  | **Object** | 35 | 62 | 29 | 45 | 24 | 21 | 216 | 170.45 |  |
|  |  |  | **Social** | 51 | 84 | 72 | 52 | 82 | 74 | 415 | 327.49 |  |
|  |  |  | **Object** | 41 | 49 | 38 | 45 | 36 | 23 | 232 | 183.08 |  |
|  |  |  | **Social** | 40 | 44 | 52 | 24 | 48 | 63 | 271 | 213.86 |  |
|  |  | **PD** | **Object** | 14 | 29 | 11 | 23 | 24 | 21 | 122 | 96.28 | P = 0.269 |
|  |  |  | **Social** | 13 | 18 | 12 | 15 | 15 | 78 | 151 | 119.16 |  |
|  |  |  | **Object** | 7 | 31 | 19 | 32 | 9 | 26 | 124 | 97.85 |  |
|  |  |  | **Social** | 41 | 42 | 53 | 44 | 20 | 26 | 226 | 178.35 |  |
|  |  |  | **Object** | 53 | 71 | 87 | 48 | 54 | 68 | 381 | 300.66 |  |
|  |  |  | **Social** | 30 | 13 | 21 | 19 | 21 | 20 | 124 | 97.85 |  |
|  |  |  | **Object** | 24 | 8 | 32 | 16 | 41 | 8 | 129 | 101.8 |  |
|  |  |  | **Social** | 24 | 23 | 41 | 18 | 23 | 16 | 145 | 114.43 |  |
|  |  |  | **Object** | 71 | 63 | 53 | 62 | 45 | 56 | 350 | 276.2 |  |
|  |  |  | **Social** | 24 | 22 | 8 | 26 | 25 | 20 | 125 | 98.64 |  |
|  |  | **PC vs. PD** |  | | | | | | | | **Object** : P = 0.648  **Social** : P < 0.01 |  |
|  | **Female** | **PC** | **Object** | 37 | 44 | 59 | 61 | 53 | 79 | 333 | 262.78 | P < 0.01 |
|  |  |  | **Social** | 45 | 29 | 45 | 54 | 53 | 42 | 268 | 211.49 |  |
|  |  |  | **Object** | 3 | 45 | 33 | 18 | 24 | 9 | 132 | 104.17 |  |
|  |  |  | **Social** | 75 | 62 | 57 | 71 | 58 | 64 | 387 | 305.4 |  |
|  |  |  | **Object** | 20 | 3 | 25 | 31 | 45 | 48 | 172 | 135.73 |  |
|  |  |  | **Social** | 43 | 52 | 67 | 74 | 42 | 81 | 359 | 283.3 |  |
|  |  |  | **Object** | 6 | 32 | 44 | 25 | 13 | 14 | 134 | 105.74 |  |
|  |  |  | **Social** | 56 | 37 | 56 | 72 | 65 | 63 | 349 | 275.41 |  |
|  |  |  | **Object** | 5 | 12 | 21 | 26 | 32 | 28 | 124 | 97.85 |  |
|  |  |  | **Social** | 64 | 57 | 72 | 36 | 54 | 75 | 358 | 282.51 |  |
|  |  | **PD** | **Object** | 43 | 49 | 4 | 16 | 7 | 13 | 132 | 104.17 | P = 0.326 |
|  |  |  | **Social** | 32 | 73 | 84 | 40 | 48 | 63 | 340 | 268.31 |  |
|  |  |  | **Object** | 56 | 75 | 67 | 65 | 50 | 10 | 323 | 254.89 |  |
|  |  |  | **Social** | 68 | 54 | 54 | 65 | 76 | 89 | 406 | 320.39 |  |
|  |  |  | **Object** | 54 | 56 | 32 | 57 | 24 | 22 | 245 | 193.34 |  |
|  |  |  | **Social** | 14 | 22 | 20 | 59 | 23 | 26 | 164 | 129.42 |  |
|  |  |  | **Object** | 6 | 41 | 35 | 17 | 14 | 22 | 135 | 106.53 |  |
|  |  |  | **Social** | 31 | 20 | 19 | 54 | 34 | 21 | 179 | 141.26 |  |
|  |  |  | **Object** | 64 | 38 | 72 | 54 | 46 | 18 | 292 | 230.43 |  |
|  |  |  | **Social** | 52 | 42 | 36 | 87 | 64 | 28 | 309 | 243.84 |  |
|  |  | **PC vs. PD** |  | | | | | | | | **Object** : P = 0.398  **Social** : P = 0.244 |  |
| **Core** | **Male** | **PC** | **Object** | 41 | 44 | 38 | 46 | 67 | 42 | 278 | 219.38 | P > 0.05 |
|  |  |  | **Social** | 17 | 40 | 58 | 54 | 34 | 47 | 250 | 197.29 |  |
|  |  |  | **Object** | 29 | 24 | 13 | 36 | 18 | 25 | 145 | 114.43 |  |
|  |  |  | **Social** | 25 | 45 | 28 | 32 | 40 | 12 | 182 | 143.62 |  |
|  |  |  | **Object** | 15 | 24 | 28 | 17 | 27 | 20 | 131 | 103.38 |  |
|  |  |  | **Social** | 22 | 18 | 24 | 9 | 22 | 25 | 120 | 94.7 |  |
|  |  |  | **Object** | 21 | 34 | 21 | 42 | 19 | 12 | 149 | 117.58 |  |
|  |  |  | **Social** | 10 | 36 | 38 | 42 | 21 | 25 | 172 | 135.73 |  |
|  |  |  | **Object** | 31 | 23 | 15 | 21 | 13 | 42 | 145 | 114.43 |  |
|  |  |  | **Social** | 20 | 16 | 32 | 18 | 15 | 24 | 125 | 98.64 |  |
|  |  | **PD** | **Object** | 6 | 8 | 7 | 6 | 3 | 5 | 35 | 27.62 | P > 0.05 |
|  |  |  | **Social** | 4 | 3 | 31 | 19 | 19 | 61 | 137 | 108.11 |  |
|  |  |  | **Object** | 8 | 3 | 2 | 0 | 4 | 2 | 19 | 14.99 |  |
|  |  |  | **Social** | 36 | 36 | 45 | 33 | 24 | 33 | 207 | 163.35 |  |
|  |  |  | **Object** | 27 | 29 | 25 | 34 | 52 | 47 | 214 | 168.88 |  |
|  |  |  | **Social** | 19 | 12 | 21 | 24 | 11 | 13 | 100 | 78.91 |  |
|  |  |  | **Object** | 9 | 5 | 3 | 5 | 6 | 8 | 36 | 28.41 |  |
|  |  |  | **Social** | 27 | 8 | 12 | 23 | 9 | 14 | 93 | 73.39 |  |
|  |  |  | **Object** | 35 | 43 | 34 | 24 | 33 | 24 | 193 | 152.3 |  |
|  |  |  | **Social** | 36 | 31 | 35 | 38 | 34 | 29 | 203 | 160.2 |  |
|  |  | **PC vs. PD** |  | | | | | | | | **Object** : P > 0.05  **Social** : P > 0.05 |  |
|  | **Female** | **PC** | **Object** | 23 | 15 | 22 | 22 | 46 | 15 | 143 | 112.85 | P > 0.05 |
|  |  |  | **Social** | 10 | 36 | 17 | 11 | 10 | 9 | 93 | 73.39 |  |
|  |  |  | **Object** | 7 | 8 | 14 | 15 | 17 | 13 | 74 | 58.4 |  |
|  |  |  | **Social** | 19 | 45 | 21 | 20 | 27 | 30 | 162 | 127.84 |  |
|  |  |  | **Object** | 18 | 2 | 10 | 3 | 5 | 5 | 43 | 33.93 |  |
|  |  |  | **Social** | 3 | 17 | 14 | 12 | 40 | 42 | 128 | 101.01 |  |
|  |  |  | **Object** | 22 | 12 | 31 | 14 | 32 | 16 | 127 | 100.22 |  |
|  |  |  | **Social** | 26 | 24 | 14 | 27 | 31 | 24 | 146 | 115.21 |  |
|  |  |  | **Object** | 11 | 14 | 12 | 13 | 10 | 12 | 72 | 56.82 |  |
|  |  |  | **Social** | 7 | 12 | 15 | 27 | 13 | 6 | 80 | 63.13 |  |
|  |  | **PD** | **Object** | 16 | 12 | 6 | 3 | 4 | 19 | 60 | 47.35 | P > 0.05 |
|  |  |  | **Social** | 7 | 6 | 15 | 16 | 23 | 27 | 94 | 74.18 |  |
|  |  |  | **Object** | 15 | 40 | 26 | 36 | 37 | 19 | 173 | 136.52 |  |
|  |  |  | **Social** | 7 | 22 | 33 | 24 | 25 | 25 | 136 | 107.32 |  |
|  |  |  | **Object** | 5 | 8 | 5 | 4 | 9 | 10 | 41 | 32.35 |  |
|  |  |  | **Social** | 2 | 2 | 8 | 11 | 20 | 9 | 52 | 41.04 |  |
|  |  |  | **Object** | 21 | 34 | 32 | 31 | 51 | 12 | 181 | 142.83 |  |
|  |  |  | **Social** | 24 | 31 | 14 | 13 | 15 | 16 | 113 | 89.17 |  |
|  |  |  | **Object** | 20 | 13 | 7 | 6 | 3 | 24 | 73 | 57.61 |  |
|  |  |  | **Social** | 16 | 12 | 42 | 31 | 36 | 32 | 169 | 133.36 |  |
|  |  | **PC vs. PD** |  | | | | | | | | **Object** : P > 0.05  **Social** : P > 0.05 |  |
