# Supplementary material for: Increased anxiety and decreased sociability induced by paternal deprivation involve the PVN-PrL OTergic pathway
Source: eLife. 2019 May 14;8:e44026. doi: 10.7554/eLife.44026 (PMC6516825; doi:10.7554/eLife.44026)
Supplement: Figure 4—source data 1. [file elife-44026-fig4-data1.doc]

**Source Data 4F**

|  |  | **Male** | | | |  | **Female** | | | |
| --- | --- | --- | --- | --- | --- | --- | --- | --- | --- | --- |
| **Nucleus** | **Treatment** | **First section (#/mm^2^)** | **Second section (#/mm^2^)** | **Third**  **Section (#/mm^2^)** | **Mean**  **(#/mm^2^)** | **Male vs. Female** | **Mean (#/mm^2^)** | **First section (#/mm^2^)** | **Second section (#/mm^2^)** | **Third**  **Section (#/mm^2^)** |
| **PVN** | **PC** | 32.517 | 93.382 | 43.356 | 56.42 | **P < 0.01** | 89.49 | 123.398 | 114.226 | 30.849 |
|  |  | 65.868 | 85.878 | 33.351 | 61.70 |  | 93.94 | 88.379 | 138.405 | 55.029 |
|  |  | 53.361 | 95.883 | 30.016 | 59.75 |  | 73.37 | 67.535 | 116.727 | 35.852 |
|  |  | 44.190 | 89.213 | 20.844 | 51.42 |  | 77.82 | 68.369 | 121.730 | 43.356 |
|  | **PD** | 28.348 | 57.530 | 18.343 | 34.74 | **P = 0.263** | 38.91 | 8.338 | 90.881 | 17.509 |
|  |  | 31.683 | 47.525 | 12.507 | 30.57 |  | 38.63 | 26.681 | 73.372 | 15.842 |
|  |  | 22.512 | 68.369 | 9.171 | 33.35 |  | 26.68 | 10.839 | 45.857 | 23.345 |
|  |  | 23.345 | 45.857 | 24.179 | 31.13 |  | 49.19 | 40.021 | 75.873 | 31.683 |
|  | **PC vs. PD** | **P < 0.01** | | | |  | **P < 0.01** | | | |
