# Supplementary material for: Increased anxiety and decreased sociability induced by paternal deprivation involve the PVN-PrL OTergic pathway
Source: eLife. 2019 May 14;8:e44026. doi: 10.7554/eLife.44026 (PMC6516825; doi:10.7554/eLife.44026)
Supplement: Figure 5—source data 1. [file elife-44026-fig5-data1.doc]

**Source Data 5**

|  |  | **Male** | | | **Female** | | |
| --- | --- | --- | --- | --- | --- | --- | --- |
| **Nucleus** | **Treatment** | **OTR** | **Tubulin** | **OTR/ Tubulin** | **OTR** | **Tubulin** | **OTR/ Tubulin** |
| **mPFC** | **PC** | 0.089 | 0.631 | 0.14 | 0.232 | 0.341 | 0.68 |
|  |  | 0.251 | 0.556 | 0.45 | 0.161 | 0.303 | 0.53 |
|  |  | 0.27 | 0.565 | 0.48 | 0.141 | 0.445 | 0.32 |
|  |  | 0.265 | 0.558 | 0.47 | 0.178 | 0.483 | 0.37 |
|  |  | 0.121 | 0.398 | 0.30 | 0.116 | 0.177 | 0.66 |
|  |  | 0.205 | 0.444 | 0.46 | 0.101 | 0.227 | 0.44 |
|  | **PD** | 0.02 | 0.401 | 0.05 | 0.038 | 0.411 | 0.09 |
|  |  | 0.018 | 0.601 | 0.03 | 0.14 | 0.341 | 0.41 |
|  |  | 0.155 | 0.545 | 0.28 | 0.024 | 0.299 | 0.08 |
|  |  | 0.016 | 0.548 | 0.03 | 0.031 | 0.336 | 0.09 |
|  |  | 0.104 | 0.457 | 0.23 | 0.026 | 0.245 | 0.11 |
|  |  | 0.046 | 0.469 | 0.10 | 0.035 | 0.25 | 0.14 |
|  | **PC vs. PD** |  |  | **P < 0.01** |  |  | **P < 0.01** |
| **NAc** | **PC** | 0.32 | 0.625 | 0.51 | 0.069 | 0.213 | 0.32 |
|  |  | 0.033 | 0.571 | 0.06 | 0.356 | 0.269 | 1.32 |
|  |  | 0.242 | 0.901 | 0.27 | 0.098 | 0.451 | 0.22 |
|  |  | 0.065 | 0.386 | 0.17 | 0.048 | 0.513 | 0.09 |
|  |  | 0.186 | 0.522 | 0.36 | 0.101 | 0.19 | 0.53 |
|  |  | 0.155 | 0.465 | 0.33 | 0.08 | 0.164 | 0.49 |
|  | **PD** | 0.035 | 0.607 | 0.06 | 0.081 | 0.274 | 0.30 |
|  |  | 0.137 | 0.684 | 0.20 | 0.133 | 0.264 | 0.50 |
|  |  | 0.297 | 0.672 | 0.44 | 0.26 | 0.227 | 1.15 |
|  |  | 0.018 | 0.559 | 0.03 | 0.098 | 0.255 | 0.38 |
|  |  | 0.172 | 0.574 | 0.30 | 0.112 | 0.192 | 0.58 |
|  |  | 0.180 | 0.541 | 0.33 | 0.132 | 0.205 | 0.64 |
|  | **PC vs. PD** |  |  | **P = 0.563** |  |  | **P = 0.665** |
| **PVN** | **PC** | 0.035 | 0.524 | 0.07 | 0.074 | 0.223 | 0.33 |
|  |  | 0.139 | 0.492 | 0.28 | 0.013 | 0.172 | 0.08 |
|  |  | 0.019 | 0.15 | 0.13 | 0.028 | 0.203 | 0.14 |
|  |  | 0.045 | 0.25 | 0.18 | 0.073 | 0.228 | 0.32 |
|  |  | 0.032 | 0.294 | 0.11 | 0.06 | 0.231 | 0.26 |
|  |  | 0.044 | 0.274 | 0.16 | 0.047 | 0.207 | 0.23 |
|  | **PD** | 0.032 | 0.549 | 0.06 | 0.069 | 0.311 | 0.22 |
|  |  | 0.15 | 0.265 | 0.57 | 0.078 | 0.206 | 0.38 |
|  |  | 0.015 | 0.247 | 0.06 | 0.085 | 0.115 | 0.74 |
|  |  | 0.077 | 0.229 | 0.34 | 0.018 | 0.08 | 0.23 |
|  |  | 0.026 | 0.308 | 0.08 | 0.041 | 0.22 | 0.19 |
|  |  | 0.028 | 0.283 | 0.10 | 0.06 | 0.23 | 0.26 |
|  | **PC vs. PD** |  |  | **P = 0.616** |  |  | **P = 0.273** |
|  |  | **V1aR** | **Tubulin** | **V1aR / Tubulin** | **V1aR** | **Tubulin** | **V1aR / Tubulin** |
| **mPFC** | **PC** | 0.028 | 0.569 | 0.05 | 0.056 | 0.516 | 0.11 |
|  |  | 0.036 | 0.802 | 0.04 | 0.068 | 0.499 | 0.14 |
|  |  | 0.041 | 0.835 | 0.05 | 0.048 | 0.451 | 0.11 |
|  |  | 0.042 | 0.743 | 0.06 | 0.033 | 0.257 | 0.13 |
|  |  | 0.03 | 0.233 | 0.13 | 0.068 | 0.345 | 0.20 |
|  |  | 0.011 | 0.254 | 0.04 | 0.063 | 0.335 | 0.19 |
|  | **PD** | 0.035 | 0.739 | 0.05 | 0.059 | 0.567 | 0.10 |
|  |  | 0.017 | 0.837 | 0.02 | 0.041 | 0.383 | 0.11 |
|  |  | 0.011 | 0.769 | 0.01 | 0.088 | 0.342 | 0.26 |
|  |  | 0.024 | 0.762 | 0.03 | 0.046 | 0.167 | 0.28 |
|  |  | 0.008 | 0.288 | 0.03 | 0.046 | 0.317 | 0.15 |
|  |  | 0.009 | 0.328 | 0.03 | 0.03 | 0.309 | 0.10 |
|  | **PC vs. PD** |  |  | **P < 0.05** |  |  | **P = 0.594** |
| **NAc** | **PC** | 0.082 | 0.471 | 0.17 | 0.072 | 0.59 | 0.12 |
|  |  | 0.042 | 0.373 | 0.11 | 0.087 | 0.589 | 0.15 |
|  |  | 0.072 | 0.45 | 0.16 | 0.088 | 0.468 | 0.19 |
|  |  | 0.01 | 0.454 | 0.02 | 0.169 | 0.57 | 0.30 |
|  |  | 0.047 | 0.352 | 0.13 | 0.066 | 0.399 | 0.17 |
|  |  | 0.049 | 0.335 | 0.15 | 0.057 | 0.377 | 0.15 |
|  | **PD** | 0.082 | 0.431 | 0.19 | 0.087 | 0.615 | 0.14 |
|  |  | 0.071 | 0.417 | 0.17 | 0.088 | 0.542 | 0.16 |
|  |  | 0.074 | 0.48 | 0.15 | 0.13 | 0.502 | 0.26 |
|  |  | 0.102 | 0.486 | 0.21 | 0.196 | 0.598 | 0.33 |
|  |  | 0.051 | 0.38 | 0.13 | 0.062 | 0.395 | 0.16 |
|  |  | 0.058 | 0.348 | 0.17 | 0.06 | 0.424 | 0.14 |
|  | **PC vs. PD** |  |  | **P = 0.092** |  |  | **P = 0.636** |
| **PVN** | **PC** | 0.079 | 0.509 | 0.16 | 0.048 | 0.445 | 0.11 |
|  |  | 0.074 | 0.489 | 0.15 | 0.042 | 0.486 | 0.09 |
|  |  | 0.108 | 0.345 | 0.31 | 0.029 | 0.434 | 0.07 |
|  |  | 0.116 | 0.384 | 0.30 | 0.04 | 0.455 | 0.09 |
|  |  | 0.098 | 0.456 | 0.21 | 0.104 | 0.774 | 0.13 |
|  |  | 0.106 | 0.444 | 0.24 | 0.089 | 0.792 | 0.11 |
|  | **PD** | 0.053 | 0.534 | 0.10 | 0.045 | 0.544 | 0.08 |
|  |  | 0.055 | 0.336 | 0.16 | 0.034 | 0.476 | 0.07 |
|  |  | 0.079 | 0.326 | 0.24 | 0.037 | 0.395 | 0.09 |
|  |  | 0.063 | 0.326 | 0.19 | 0.018 | 0.312 | 0.06 |
|  |  | 0.085 | 0.437 | 0.19 | 0.09 | 0.778 | 0.12 |
|  |  | 0.074 | 0.4 | 0.19 | 0.108 | 0.71 | 0.15 |
|  | **PC vs. PD** |  |  | **P = 0.179** |  |  | **P = 0.830** |
|  |  | **AVP** | **Tubulin** | **AVP / Tubulin** | **AVP** | **Tubulin** | **AVP / Tubulin** |
| **mPFC** | **PC** | 0.304 | 0.412 | 0.74 | 0.166 | 0.497 | 0.33 |
|  |  | 0.287 | 0.443 | 0.65 | 0.139 | 0.502 | 0.28 |
|  |  | 0.37 | 0.464 | 0.80 | 0.27 | 0.448 | 0.60 |
|  |  | 0.365 | 0.439 | 0.83 | 0.306 | 0.264 | 1.16 |
|  |  | 0.298 | 0.397 | 0.75 | 0.267 | 0.274 | 0.97 |
|  |  | 0.272 | 0.402 | 0.68 | 0.223 | 0.279 | 0.80 |
|  | **PD** | 0.254 | 0.421 | 0.60 | 0.161 | 0.531 | 0.30 |
|  |  | 0.34 | 0.382 | 0.89 | 0.181 | 0.383 | 0.47 |
|  |  | 0.375 | 0.412 | 0.91 | 0.282 | 0.331 | 0.85 |
|  |  | 0.301 | 0.305 | 0.99 | 0.27 | 0.163 | 1.66 |
|  |  | 0.28 | 0.378 | 0.74 | 0.204 | 0.187 | 1.09 |
|  |  | 0.284 | 0.368 | 0.77 | 0.211 | 0.204 | 1.03 |
|  | **PC vs. PD** |  |  | **P = 0.253** |  |  | **P = 0.408** |
| **NAc** | **PC** | 0.192 | 0.471 | 0.41 | 0.303 | 0.59 | 0.51 |
|  |  | 0.034 | 0.373 | 0.09 | 0.266 | 0.589 | 0.45 |
|  |  | 0.095 | 0.45 | 0.21 | 0.209 | 0.468 | 0.45 |
|  |  | 0.119 | 0.454 | 0.26 | 0.184 | 0.57 | 0.32 |
|  |  | 0.093 | 0.404 | 0.23 | 0.267 | 0.443 | 0.60 |
|  |  | 0.093 | 0.372 | 0.25 | 0.223 | 0.344 | 0.65 |
|  | **PD** | 0.141 | 0.431 | 0.33 | 0.305 | 0.615 | 0.50 |
|  |  | 0.085 | 0.417 | 0.20 | 0.241 | 0.542 | 0.44 |
|  |  | 0.158 | 0.48 | 0.33 | 0.174 | 0.502 | 0.35 |
|  |  | 0.19 | 0.486 | 0.39 | 0.197 | 0.598 | 0.33 |
|  |  | 0.109 | 0.339 | 0.32 | 0.204 | 0.395 | 0.52 |
|  |  | 0.115 | 0.347 | 0.33 | 0.211 | 0.38 | 0.56 |
|  | **PC vs. PD** |  |  | **P = 0.151** |  |  | **P = 0.437** |
| **PVN** | **PC** | 0.402 | 0.499 | 0.81 | 0.142 | 0.44 | 0.32 |
|  |  | 0.415 | 0.465 | 0.89 | 0.111 | 0.475 | 0.23 |
|  |  | 0.376 | 0.339 | 1.11 | 0.101 | 0.42 | 0.24 |
|  |  | 0.434 | 0.381 | 1.14 | 0.191 | 0.44 | 0.43 |
|  |  | 0.284 | 0.342 | 0.83 | 0.155 | 0.389 | 0.40 |
|  |  | 0.298 | 0.405 | 0.74 | 0.158 | 0.335 | 0.47 |
|  | **PD** | 0.383 | 0.499 | 0.77 | 0.098 | 0.53 | 0.18 |
|  |  | 0.379 | 0.317 | 1.20 | 0.102 | 0.464 | 0.22 |
|  |  | 0.392 | 0.329 | 1.19 | 0.129 | 0.383 | 0.34 |
|  |  | 0.274 | 0.274 | 1.00 | 0.118 | 0.294 | 0.4 |
|  |  | 0.254 | 0.319 | 0.80 | 0.114 | 0.317 | 0.36 |
|  |  | 0.333 | 0.275 | 1.21 | 0.131 | 0.29 | 0.45 |
|  | **PC vs. PD** |  |  | **P = 0.340** |  |  | **P = 0.687** |
