# Supplementary material for: Increased anxiety and decreased sociability induced by paternal deprivation involve the PVN-PrL OTergic pathway
Source: eLife. 2019 May 14;8:e44026. doi: 10.7554/eLife.44026 (PMC6516825; doi:10.7554/eLife.44026)
Supplement: Figure 6—source data 1. [file elife-44026-fig6-data1.doc]

**Source Data 6E, F, H, I**

| **Sex** | **Treatment** | **Time in the central area (%)** | **Multiple comparisons** | **Total distance (cm)** | **Multiple comparisons** |
| --- | --- | --- | --- | --- | --- |
| **Male** | **CSF** | 10.13 | CSF vs. 10ng OT **(P < 0.05)**;  CSF vs. 10ng OT/10ng OTA **(P < 0.05)**. | 2151.91 | **n.s.** |
|  |  | 7.42 |  | 2104.78 |  |
|  |  | 4.04 |  | 968.02 |  |
|  |  | 11.67 |  | 2215 |  |
|  |  | 4.98 |  | 1471.47 |  |
|  |  | 7.75 |  | 1863.97 |  |
|  | **1ng OT** | 11.69 | 1ng OT vs. 10ng OT **(P < 0.05)**;  1ng OT vs. 10ng OT/10ng OTA **(P < 0.05)**. | 1903.38 | **n.s.** |
|  |  | 5.87 |  | 1714.09 |  |
|  |  | 4.73 |  | 1266.46 |  |
|  |  | 15.4 |  | 2357.25 |  |
|  |  | 6.99 |  | 1040.1 |  |
|  |  | 1.25 |  | 1440.3 |  |
|  | **10ng OT** | 16.29 | 10ng OT vs. CSF**(P < 0.05)**;  10ng OT vs. 1ng OT **(P < 0.05)**;  10ng OT vs. 10ng OT/100ng OTA **(P < 0.05)**;  10ng OT vs. 10ng OT/10ng V1aRA **(P < 0.05)**. | 2708.45 | **n.s.** |
|  |  | 18.43 |  | 2340.55 |  |
|  |  | 19.91 |  | 1867.77 |  |
|  |  | 12.98 |  | 1798.74 |  |
|  |  | 14.06 |  | 1453.51 |  |
|  |  | 11.25 |  | 1925.63 |  |
|  | **10ng OT/10ng OTA** | 10.32 | 10ng OT/10ng OTA vs. CSF **(P < 0.05)**;  10ng OT/10ng OTA vs. 1ng OT **(P < 0.05)**;  10ng OT/10ng OTA vs. 10ng OT/100ng OTA **(P < 0.05)**;  10ng OT/10ng OTA vs. 10ng OT/10ng V1aRA **(P < 0.05)**. | 1924.31 | **n.s.** |
|  |  | 18.71 |  | 1871.24 |  |
|  |  | 13.24 |  | 2340.45 |  |
|  |  | 14.56 |  | 1920.64 |  |
|  |  | 17.23 |  | 2534.21 |  |
|  |  | 12.25 |  | 1534.65 |  |
|  | **10ng OT/100ng OTA** | 6.71 | 10ng OT/100ng OTA vs. 10ng OT (**P < 0.05**);  10ng OT/100ng OTA vs. 10ng OT/10ng OTA **(P < 0.05)**. | 2647.7 | **n.s.** |
|  |  | 2.07 |  | 2308.72 |  |
|  |  | 9.39 |  | 3107.84 |  |
|  |  | 11.96 |  | 1985.38 |  |
|  |  | 6.35 |  | 1317.71 |  |
|  | **10ng OT/10ng V1aRA** | 10.80 | 10ng OT/10ng V1aRA vs. 10ng OT **(P < 0.05)**;  10ng OT/10ng V1aRA vs. 10ng OT/10ng OTA **(P < 0.05)**. | 2266.31 | **n.s.** |
|  |  | 6.92 |  | 1771.8 |  |
|  |  | 5.95 |  | 2550.23 |  |
|  |  | 7.24 |  | 1823.11 |  |
|  |  | 11.10 |  | 2492.49 |  |
|  |  | 7.30 |  | 3392.35 |  |
| **Female** | **CSF** | 6.39 | CSF vs. 1ng OT **(P < 0.05)**;  CSF vs. 10ng OT **(P < 0.05)**;  CSF vs. 10ng OT/10ng V1aRA **(P < 0.05)**. | 2334.3 | **n.s.** |
|  |  | 4.3 |  | 2092.71 |  |
|  |  | 1.05 |  | 2581.82 |  |
|  |  | 2.38 |  | 919.84 |  |
|  |  | 0.78 |  | 947.82 |  |
|  |  | 10.5 |  | 2944.32 |  |
|  | **1ng OT** | 10.06 | 1ng OT vs. CSF **(P < 0.05)**;  1ng OT vs. 1ng OT/10ng OTA **(P < 0.05)**;  1ng OT vs. 1ng OT/100ng OTA **(P < 0.05)**. | 2759.88 | **n.s.** |
|  |  | 10.53 |  | 1907.58 |  |
|  |  | 19.76 |  | 1726.71 |  |
|  |  | 10.79 |  | 2033.75 |  |
|  |  | 6.93 |  | 1981.8 |  |
|  |  | 10.69 |  | 2012.34 |  |
|  | **10ng OT** | 12.9 | 10ng OT vs. CSF (**P < 0.05)**;  10ng OT vs. 1ng OT/10ng OTA **(P < 0.05)**;  10ng OT vs. 1ng OT/100ng OTA **(P < 0.05)**. | 2565.26 | **n.s.** |
|  |  | 11.27 |  | 2372.54 |  |
|  |  | 13.19 |  | 939.31 |  |
|  |  | 13 |  | 2028.37 |  |
|  |  | 7.18 |  | 1613.2 |  |
|  |  | 18.71 |  | 1525.94 |  |
|  | **1ng OT/10ng OTA** | 0.72 | 1ng OT/10ng OTA vs. 1ng OT **(P < 0.05)**;  1ng OT/10ng OTA vs. 10ng OT **(P < 0.05)**;  1ng OT/10ng OTA vs. 10ng OT/10ng V1aRA **(P < 0.05)**. | 1118.02 | **n.s.** |
|  |  | 6.94 |  | 1016.35 |  |
|  |  | 4.43 |  | 1401.86 |  |
|  |  | 6.65 |  | 1717.68 |  |
|  |  | 4.96 |  | 680.64 |  |
|  | **1ng OT/100ng OTA** | 4.97 | 1ng OT/100ng OTA vs. 1ng OT **(P < 0.05)**;  1ng OT/100ng OTA vs. 10ng OT **(P < 0.05)**;  1ng OT/100ng OTA vs. 10ng OT/10ng V1aRA **(P < 0.05)**. | 2294.93 | **n.s.** |
|  |  | 5.09 |  | 1075.42 |  |
|  |  | 2.09 |  | 2635.12 |  |
|  |  | 7.8 |  | 1937.61 |  |
|  |  | 4.76 |  | 1694.51 |  |
|  | **10ng OT/10ng V1aRA** | 9.58 | 10ng OT/10ng V1aRA vs. CSF (**P < 0.05)**;  10ng OT/10ng V1aRA vs. 1ng OT/10ng OTA **(P < 0.05)**  10ng OT/10ng V1aRA vs. 1ng OT/100ng OTA **(P < 0.05)** | 1426.1 | **n.s.** |
|  |  | 14.01 |  | 1076.65 |  |
|  |  | 9.29 |  | 3386.96 |  |
|  |  | 21.45 |  | 1213.67 |  |
|  |  | 15 |  | 851.46 |  |
|  |  | 10.33 |  | 1868.91 |  |

**Source Data 6G, J**

| **Sex** | **Treatment** | **Object (%)** | **Social (%)** | **Object vs. Social** |
| --- | --- | --- | --- | --- |
| **Male** | **CSF** | 52.71 | 83.42 | **P = 0.348** |
|  |  | 43.69 | 51.81 |  |
|  |  | 11.1 | 37.09 |  |
|  |  | 52.55 | 47.15 |  |
|  |  | 31.85 | 11.25 |  |
|  |  | 35.02 | 44.85 |  |
|  | **1ng OT** | 55.68 | 53.08 | **P = 0.054** |
|  |  | 27.46 | 79.31 |  |
|  |  | 47.44 | 74.86 |  |
|  |  | 57.95 | 97.45 |  |
|  |  | 46.72 | 47.93 |  |
|  |  | 57.77 | 72.65 |  |
|  | **10ng OT** | 37.45 | 59.29 | **P < 0.0083** |
|  |  | 60.23 | 74.15 |  |
|  |  | 35.31 | 79.89 |  |
|  |  | 59.6 | 69.68 |  |
|  |  | 38.24 | 58.84 |  |
|  |  | 62.34 | 87.52 |  |
|  | **10ng OT/10ng OTA** | 25.34 | 55.28 | **P < 0. 0.0083** |
|  |  | 35.13 | 65.32 |  |
|  |  | 40.23 | 71.26 |  |
|  |  | 42.34 | 58.23 |  |
|  |  | 37.89 | 57.42 |  |
|  |  | 49.32 | 61.47 |  |
|  | **10ng OT/100g OTA** | 49.49 | 72.44 | **P = 0.304** |
|  |  | 53 | 77.01 |  |
|  |  | 28.97 | 45.02 |  |
|  |  | 47.22 | 76.96 |  |
|  |  | 68.23 | 38.77 |  |
|  | **10ng OT/10ng V1aRA** | 39.69 | 45.57 | **P = 0.805** |
|  |  | 45.85 | 47.24 |  |
|  |  | 89.26 | 75.64 |  |
|  |  | 43.54 | 33.86 |  |
|  |  | 32.2 | 52.67 |  |
|  |  | 31.1 | 34.38 |  |
| **Female** | **CSF** | 46.23 | 70.48 | **P = 0.175** |
|  |  | 35.82 | 54.3 |  |
|  |  | 18.77 | 34.81 |  |
|  |  | 22.43 | 69 |  |
|  |  | 55.97 | 43.64 |  |
|  |  | 44.03 | 35.57 |  |
|  | **1ng OT** | 13.47 | 65.43 | **P < 0.0083** |
|  |  | 54.84 | 67.12 |  |
|  |  | 42.97 | 63.25 |  |
|  |  | 38.85 | 68.28 |  |
|  |  | 23.02 | 68.09 |  |
|  |  | 21.82 | 69.09 |  |
|  | **10ng OT** | 48.24 | 58.84 | **P < 0.0083** |
|  |  | 25.49 | 57.3 |  |
|  |  | 39.7 | 90.18 |  |
|  |  | 81.42 | 99.73 |  |
|  |  | 43.37 | 80.2 |  |
|  |  | 38.53 | 67.57 |  |
|  | **1ng OT/10ng OTA** | 17.42 | 58.17 | **P = 0.301** |
|  |  | 53.18 | 78.83 |  |
|  |  | 60.26 | 51.88 |  |
|  |  | 43.07 | 89.15 |  |
|  |  | 40.08 | 17.01 |  |
|  | **1ng OT/100g OTA** | 42.51 | 60.58 | **P = 0.300** |
|  |  | 26.93 | 16.89 |  |
|  |  | 60.77 | 65.85 |  |
|  |  | 36.44 | 44.57 |  |
|  |  | 27.95 | 33.53 |  |
|  | **10ng OT/10ng V1aRA** | 23.63 | 67.21 | **P < 0.0083** |
|  |  | 17.94 | 65.9 |  |
|  |  | 27.08 | 44.64 |  |
|  |  | 53.36 | 73.5 |  |
|  |  | 71.32 | 82.51 |  |
|  |  | 30.24 | 84.85 |  |
