# Supplementary material for: Increased anxiety and decreased sociability induced by paternal deprivation involve the PVN-PrL OTergic pathway
Source: eLife. 2019 May 14;8:e44026. doi: 10.7554/eLife.44026 (PMC6516825; doi:10.7554/eLife.44026)
Supplement: Figure 7—source data 1. [file elife-44026-fig7-data1.docx]

**Source Data for Figure 7H, I**

| **Sex** | **Treatment** | **First**  **section**  **(#)** | **Second**  **section**  **(#)** | **Third**  **section**  **(#)** | **Fourth**  **section**  **(#)** | **Fifth**  **section**  **(#)** | **Sixth**  **section**  **(#)** | **Total** | **Mean**  **(#/mm^2^)** | **Ctrl vs. ChR2** |
| --- | --- | --- | --- | --- | --- | --- | --- | --- | --- | --- |
| **Male** | **Ctrl** | 2 | 3 | 2 | 4 | 8 | 0 | 19 | 10.31 | **P < 0.01** |
|  |  | 2 | 1 | 2 | 2 | 4 | 1 | 12 | 6.51 |  |
|  |  | 5 | 9 | 3 | 1 | 1 | 3 | 22 | 11.94 |  |
|  |  | 1 | 3 | 4 | 1 | 1 | 1 | 11 | 5.97 |  |
|  | **ChR2** | 18 | 39 | 29 | 40 | 48 | 22 | 196 | 106.34 |  |
|  |  | 27 | 51 | 60 | 112 | 57 | 53 | 360 | 195.31 |  |
|  |  | 44 | 56 | 54 | 69 | 51 | 51 | 325 | 176.32 |  |
|  |  | 29 | 56 | 96 | 72 | 127 | 115 | 495 | 268.55 |  |
|  |  | 46 | 107 | 63 | 104 | 83 | 54 | 457 | 247.94 |  |
| **Female** | **Ctrl** | 1 | 0 | 0 | 4 | 3 | 2 | 10 | 5.43 | **P < 0.01** |
|  |  | 6 | 4 | 4 | 15 | 18 | 16 | 63 | 34.18 |  |
|  |  | 10 | 3 | 2 | 17 | 8 | 22 | 62 | 33.64 |  |
|  |  | 5 | 0 | 16 | 2 | 12 | 2 | 37 | 20.07 |  |
|  | **ChR2** | 61 | 50 | 69 | 25 | 60 | 64 | 329 | 178.49 |  |
|  |  | 49 | 82 | 64 | 73 | 61 | 70 | 399 | 216.47 |  |
|  |  | 71 | 37 | 29 | 20 | 30 | 35 | 222 | 120.44 |  |
|  |  | 30 | 62 | 47 | 24 | 66 | 62 | 291 | 157.88 |  |
|  |  | 41 | 50 | 72 | 37 | 52 | 63 | 315 | 170.9 |  |

**Source Data for Figure 7J, K**

| **Sex** | **Treatment** | **Object (%)** | **Social (%)** | **Object vs. Social** |
| --- | --- | --- | --- | --- |
| **Male** | **Ctrl** | 43.93 | 69.69 | **P = 0.113** |
|  |  | 51.45 | 46.05 |  |
|  |  | 5.72 | 35.24 |  |
|  |  | 22.51 | 30.49 |  |
|  |  | 26.63 | 19.87 |  |
|  |  | 23.56 | 62.64 |  |
|  | **ChR2** | 33.84 | 99.24 | **P < 0.025** |
|  |  | 51.63 | 62.04 |  |
|  |  | 41.79 | 62.47 |  |
|  |  | 46.42 | 75.39 |  |
|  |  | 13.15 | 58.06 |  |
|  |  | 26.79 | 61 |  |
|  | **Ctrl vs. ChR2** | **n.s.** | **n.s.** |  |
| **Female** | **Ctrl** | 42.75 | 48.29 | **P= 0.668** |
|  |  | 31.49 | 32.61 |  |
|  |  | 42.92 | 36.58 |  |
|  |  | 36.71 | 43.57 |  |
|  |  | 34.72 | 50.67 |  |
|  |  | 59.61 | 58.69 |  |
|  | **ChR2** | 42.76 | 91.95 | **P < 0.025** |
|  |  | 12.62 | 28.81 |  |
|  |  | 33.89 | 88.91 |  |
|  |  | 27.81 | 53.18 |  |
|  |  | 31.7 | 60.7 |  |
|  |  | 38.34 | 73.47 |  |
|  | **Ctrl vs. ChR2** | **n.s.** | **P < 0.025** |  |

**Source Data for Figure 7 L, M, N, O**

| **Sex** | **Treatment** | **Time in the central area (%)** | **Total distance (cm)** |
| --- | --- | --- | --- |
| **Male** | **Ctrl** | 6.47 | 1995.03 |
|  |  | 8.6 | 1763.79 |
|  |  | 2.38 | 2058.05 |
|  |  | 9.41 | 1994.4 |
|  |  | 4.77 | 2669.95 |
|  |  | 8.83 | 2164.63 |
|  | **ChR2** | 21.1 | 3053.72 |
|  |  | 11.21 | 1610.61 |
|  |  | 11.89 | 1676.94 |
|  |  | 13.88 | 2259.38 |
|  |  | 17.6 | 2524.34 |
|  |  | 19.13 | 1297.88 |
|  | **Ctrl vs. ChR2** | **P < 0.01** | **P = 0.903** |
| **Female** | **Ctrl** | 6.1 | 2713.56 |
|  |  | 2.78 | 2367.09 |
|  |  | 0.86 | 2425.18 |
|  |  | 6.49 | 2276.32 |
|  |  | 2.88 | 1651.84 |
|  |  | 13.7 | 1114.4 |
|  | **ChR2** | 25.03 | 1542.26 |
|  |  | 16.32 | 1103.16 |
|  |  | 20.36 | 1674.54 |
|  |  | 13.05 | 2839.62 |
|  |  | 17.31 | 1054.14 |
|  |  | 10.08 | 2428.07 |
|  | **Ctrl vs. ChR2** | **P < 0.01** | **P= 0.424** |
