# Supplementary material for: Increased anxiety and decreased sociability induced by paternal deprivation involve the PVN-PrL OTergic pathway
Source: eLife. 2019 May 14;8:e44026. doi: 10.7554/eLife.44026 (PMC6516825; doi:10.7554/eLife.44026)
Supplement: Figure 8—source data 1. [file elife-44026-fig8-data1.docx]

**Source Data for Figure 8H, I**

| **Sex** | **Treatment** | **First**  **section**  **(#)** | | **Second**  **section**  **(#)** | | **Third**  **section**  **(#)** | | **Fourth**  **section**  **(#)** | | **Fifth**  **section**  **(#)** | | **Sixth**  **section**  **(#)** | | **Total** | **Mean**  **(#/mm^2^)** | **Ctrl vs. ChR2** |
| --- | --- | --- | --- | --- | --- | --- | --- | --- | --- | --- | --- | --- | --- | --- | --- | --- |
|  |  |  |  |  |  |  |  |  |  |  |  |  |  |  |  |  |
| **Male** | **Ctrl** | 56 | 62 | 52 | 62 | 54 | 72 | 53 | 35 | 37 | 46 | 25 | 44 | 598 | 162.22 | **P < 0.01** |
|  |  | 8 | 17 | 39 | 64 | 34 | 27 | 26 | 34 | 46 | 38 | 28 | 32 | 393 | 106.61 |  |
|  |  | 41 | 37 | 39 | 27 | 52 | 36 | 52 | 67 | 47 | 46 | 32 | 30 | 506 | 137.26 |  |
|  |  | 24 | 26 | 41 | 16 | 63 | 41 | 34 | 42 | 35 | 26 | 30 | 20 | 398 | 107.96 |  |
|  | **NpHR3.0** | 10 | 28 | 19 | 18 | 14 | 21 | 1 | 0 | 18 | 23 | 12 | 22 | 186 | 50.46 |  |
|  |  | 23 | 31 | 18 | 31 | 7 | 24 | 13 | 18 | 16 | 17 | 28 | 37 | 263 | 71.34 |  |
|  |  | 19 | 15 | 21 | 34 | 31 | 22 | 15 | 16 | 12 | 11 | 5 | 3 | 204 | 55.34 |  |
|  |  | 11 | 15 | 21 | 19 | 36 | 24 | 20 | 15 | 16 | 24 | 31 | 27 | 259 | 70.26 |  |
| **Female** | **Ctrl** | 51 | 10 | 36 | 8 | 37 | 45 | 56 | 62 | 51 | 42 | 30 | 45 | 473 | 128.31 | **P < 0.01** |
|  |  | 5 | 28 | 41 | 34 | 58 | 17 | 46 | 31 | 32 | 25 | 21 | 22 | 360 | 97.66 |  |
|  |  | 31 | 24 | 54 | 27 | 34 | 63 | 25 | 63 | 42 | 19 | 63 | 36 | 481 | 130.48 |  |
|  |  | 45 | 42 | 37 | 54 | 34 | 62 | 54 | 32 | 67 | 25 | 31 | 43 | 526 | 142.69 |  |
|  | **NpHR3.0** | 27 | 2 | 23 | 22 | 18 | 8 | 55 | 12 | 24 | 25 | 29 | 8 | 253 | 68.63 |  |
|  |  | 29 | 16 | 40 | 9 | 30 | 22 | 29 | 32 | 39 | 17 | 28 | 15 | 306 | 83.01 |  |
|  |  | 8 | 26 | 24 | 50 | 15 | 25 | 21 | 44 | 24 | 35 | 18 | 16 | 306 | 83.01 |  |
|  |  | 24 | 29 | 43 | 8 | 32 | 23 | 41 | 25 | 42 | 19 | 31 | 30 | 347 | 94.13 |  |

**Source Data for Figure 8J, K**

| **Sex** | **Treatment** | **Object (%)** | **Social (%)** | **Object vs. Social** |
| --- | --- | --- | --- | --- |
| **Male** | **Ctrl** | 45.55 | 58.95 | **P < 0.025** |
|  |  | 27.99 | 79.02 |  |
|  |  | 26.81 | 45.45 |  |
|  |  | 31.62 | 58.48 |  |
|  |  | 40.99 | 70.63 |  |
|  |  | 15.66 | 98.78 |  |
|  | **NpHR3.0** | 2.51 | 57.09 | **P = 0.154** |
|  |  | 62.81 | 64.02 |  |
|  |  | 25.32 | 34.64 |  |
|  |  | 25.87 | 49.07 |  |
|  |  | 26.39 | 38.1 |  |
|  |  | 79.89 | 70.88 |  |
|  | **Ctrl vs. NpHR3.0** | **n.s.** | **n.s.** |  |
| **Female** | **Ctrl** | 16.02 | 39.66 | **P < 0.025** |
|  |  | 16.41 | 64.83 |  |
|  |  | 32.53 | 45.69 |  |
|  |  | 25.93 | 82 |  |
|  |  | 35.25 | 51.08 |  |
|  |  | 32.41 | 56.53 |  |
|  | **NpHR3.0** | 34.75 | 23.54 | **P = 0.642** |
|  |  | 21.31 | 55.64 |  |
|  |  | 71.78 | 65.77 |  |
|  |  | 41.84 | 44.98 |  |
|  |  | 68.22 | 60.84 |  |
|  |  | 5.7 | 13.03 |  |
|  | **Ctrl vs. NpHR3.0** | **n.s.** | **n.s.** |  |

**Source Data for Figure 8 L, M, N, O**

| **Sex** | **Treatment** | **Time in the central area (%)** | **Total distance (cm)** |
| --- | --- | --- | --- |
| **Male** | **Ctrl** | 25.36 | 2727.51 |
|  |  | 39.97 | 1206.64 |
|  |  | 14.89 | 2380.04 |
|  |  | 14.59 | 2389.73 |
|  |  | 16.06 | 2836.56 |
|  |  | 21.44 | 2859.45 |
|  | **NpHR3.0** | 5.46 | 1976.67 |
|  |  | 10.42 | 3300.69 |
|  |  | 9.87 | 3850.91 |
|  |  | 14.15 | 3112.75 |
|  |  | 10.57 | 2396.77 |
|  |  | 3.38 | 2446.26 |
|  | **Ctrl vs. NpHR3.0** | **P < 0.05** | **P = 0.267** |
| **Female** | **Ctrl** | 17.58 | 2411.17 |
|  |  | 13.05 | 1841.42 |
|  |  | 22.34 | 2804.52 |
|  |  | 24.14 | 2153.36 |
|  |  | 24.57 | 3179.03 |
|  |  | 24.45 | 4869.12 |
|  | **NpHR3.0** | 12.53 | 2566.63 |
|  |  | 6.96 | 3570.07 |
|  |  | 9.16 | 3177.87 |
|  |  | 5.83 | 1286.82 |
|  |  | 8.14 | 1854.56 |
|  |  | 7.95 | 2129 |
|  | **Ctrl vs. NpHR3.0** | **P < 0.01** | **P= 0.446** |
