# Supplementary material for: Increased anxiety and decreased sociability induced by paternal deprivation involve the PVN-PrL OTergic pathway
Source: eLife. 2019 May 14;8:e44026. doi: 10.7554/eLife.44026 (PMC6516825; doi:10.7554/eLife.44026)
Supplement: Transparent reporting form [file elife-44026-transrepform.docx]

***eLife’s* transparent reporting form**

We encourage authors to provide detailed information *within their submission* to facilitate the interpretation and replication of experiments. Authors can upload supporting documentation to indicate the use of appropriate reporting guidelines for health-related research (see [EQUATOR Network](http://www.equator-network.org/%20)), life science research (see the [BioSharing Information Resource](https://biosharing.org/)), or the [ARRIVE guidelines](http://www.plosbiology.org/article/info:doi/10.1371/journal.pbio.1000412) for reporting work involving animal research. Where applicable, authors should refer to any relevant reporting standards documents in this form.

If you have any questions, please consult our Journal Policies and/or contact us: [editorial@elifesciences.org](mailto:editorial@elifesciences.org).

**Sample-size estimation**

- You should state whether an appropriate sample size was computed when the study was being designed
- You should state the statistical method of sample size computation and any required assumptions
- If no explicit power analysis was used, you should describe how you decided what sample (replicate) size (number) to use

Please outline where this information can be found within the submission (e.g., sections or figure legends), or explain why this information doesn’t apply to your submission:

**1 This information can be found in the following sections:**

**Figure 1:**

Effects of PD on anxiety-like behavior and social preference in adult mandarin voles. Effect of PD on percentage of time in the central area and total distance of mandarin voles in the open ﬁeld test (Male: PC: n = 7, PD: n = 7; Female: PC: n = 7, PD: n = 7). Independent sample t-tests (PC vs. PD), the signiﬁcance level was set at p < 0.05. Effect of PD on percentage of time in the light area of mandarin voles in the light and dark box test (Male: PC: n = 7, PD: n = 7; Female: PC: n = 7, PD: n = 7). Independent sample t-tests (PC vs. PD), the signiﬁcance level was set at p < 0.05. Effect of PD on social preference in males (PC: n = 7, PD: n = 7) and females (PC: n = 7, PD: n = 7). Two-way ANOVA (treatment × stimulus: PC vs. PD and object stimulus vs. social stimulus). Paired t-tests were used to compare approach/avoidance with Bonferroni correction for multiple comparisons, the threshold for signiﬁcance was set as p<0.025.

**Figure 2, 3 and 4:**

For each vole, six brain sections were counted for quantifying c-fos positive cells or OT/c-fos-positive cells; the number of OT-IR neurons (PVN) was also counted, three representative sections from anterior to posterior anatomically matched between subjects were chosen to minimize variability. An observer blind to experimental conditions performed the entire analysis once. Effect of PD on c-fos or OT/c-fos neurons (Male: PC: n = 5, PD: n = 5; Female: PC: n = 5, PD: n = 5). Effect of PD on OT-IR neurons in the PVN (Male: PC: n = 4, PD: n = 4; Female: PC: n = 4, PD: n = 4). Two-way ANOVA (treatment × sex: PC vs. PD and male vs. female), the signiﬁcance level was set at p<0.01.

**Figure 5:**

OTR, V1aR and AVP quantification was performed using ImageJ software, and all signals were normalized within the same membrane to β-Tubulin. Effects of PD on mesocorticolimbic OTR, V1aR and AVP immunoreactivity in male and female mandarin voles (Male: PC: n = 6, PD: n = 6; Female: PC: n = 6, PD: n = 6). Independent sample t-tests (PC vs. PD), the signiﬁcance level was set at p<0.05.

**Figure 6:**

Effects of PrL OT administration on anxiety-like behavior and social preference following preweaning paternal deprivation in adult mandarin voles. Percentage of time in the central area and total distance in the open field test (Male: CSF: n = 6, 1ng OT: n = 6, 10ng OT: n = 6, 10ng OT /10 ng OTA: n = 6, 10ng OT /100 ng OTA: n = 5, 10 ng OT/10 ng V1aRA = 6; Female: CSF: n = 6, 1ng OT: n = 6, 10ng OT: n = 6, 1ng OT /10 ng OTA: n = 5, 1ng OT /100 ng OTA: n = 5, 10 ng OT/10 ng V1aRA = 6). One-way ANOVA followed by SNK post-hoc tests, the signiﬁcance level was set at p<0.05. OT in the PrL promoted social preference in male (CSF: n = 6, 1ng OT: n = 6, 10ng OT: n = 6, 10ng OT /10 ng OTA: n = 6, 10ng OT /100 ng OTA: n = 5, 10 ng OT/10 ng V1aRA = 6) and female( CSF: n = 6, 1ng OT: n = 6, 10ng OT: n = 6, 1ng OT /10 ng OTA: n = 5, 1ng OT /100 ng OTA: n = 5, 10 ng OT/10 ng V1aRA = 6) voles. (treatment × sex: PC vs. PD and male vs. female). Paired t-tests were used to compare approach/avoidance with Bonferroni correction for multiple comparisons, the threshold for signiﬁcance was set as p<0.0083.

**Figure 7:**

Optogenetic stimulation of OT projection in the PrL rescued changes in emotion and social preference behavior induced by PD. Quantification of c-fos in the PrL of males (Ctrl: n = 4; ChR2: n = 5) and females (Ctrl: n = 4; ChR2: n = 5) after photostimulation, Independent sample t-tests, the signiﬁcance level was set at p<0.05. Optogenetic activation of oxytocinergic fibers in the PrL increase social preference of males (Ctrl: n = 6; ChR2: n = 6) and females (Ctrl: n = 6; ChR2: n = 6), the signiﬁcance level was set at p<0.025, Two-way ANOVA (factors: treatment × stimulus). Activation of PVN- PrL oxytocinergic projection significantly increased the percentage of time in the central area of males (Ctrl: n = 6; ChR2: n = 6) and females (Ctrl: n = 6; ChR2: n = 6), but did not influence total distance in males (Ctrl: n = 6; ChR2: n = 6) and females (Ctrl: n = 6; ChR2: n = 6), Independent sample t-tests, the signiﬁcance level was set at p<0.05.

**Figure 8:**

Optogenetic inhibition of OT projection in the PrL elicited anxiety-like behavior and attenuated social preference in naïve voles. Quantification of c-fos in the PrL of males (Ctrl: n = 4; eNpHR3.0: n = 4) and females (Ctrl: n = 4; eNpHR3.0: n = 4) after yellow light photostimulation, Independent sample t-tests, the signiﬁcance level was set at p<0.05. Optogenetic inhibition of oxytocinergic fibers in the PrL decreased social preference of males (Ctrl: n = 6; eNpHR3.0: n = 6) and females (Ctrl: n = 6; eNpHR3.0: n = 6), the signiﬁcance level was set at p<0.025, Two-way ANOVA (factors: treatment × stimulus). Inhibition of PVN- PrL oxytocinergic projection significantly decreased the percentage of time in the central area of males (Ctrl: n = 6; eNpHR3.0: n = 6) and females (Ctrl: n = 6; eNpHR3.0: n = 6), but did not influence total distance in males (Ctrl: n = 6; eNpHR3.0: n = 6) and females (Ctrl: n = 6; eNpHR3.0: n = 6), Independent sample t-tests, the signiﬁcance level was set at p<0.05.

**Replicates**

- You should report how often each experiment was performed
- You should include a definition of biological versus technical replication
- The data obtained should be provided and sufficient information should be provided to indicate the number of independent biological and/or technical replicates
- If you encountered any outliers, you should describe how these were handled
- Criteria for exclusion/inclusion of data should be clearly stated
- High-throughput sequence data should be uploaded before submission, with a private link for reviewers provided (these are available from both GEO and ArrayExpress)

Please outline where this information can be found within the submission (e.g., sections or figure legends), or explain why this information doesn’t apply to your submission:

**This information can be found in materials and methods.**

**Figure 1:**

Each animal from every group was tested only once in the open field test (OFT), Light and dark test (LDT), Social preference test (SPT) and the social preference test (SPT). And behavioral responses for each animal were scored and quantified just once.

**Figure 2, 3 and 4:**

For each vole, six brain sections were counted for quantifying c-Fos positive cells (PrL: left and right; NAcc: right) or OT/c-Fos-positive cells (PVN); the number of OT-IR neurons (PVN) were counted, three representative sections from anterior to posterior anatomically matched between subjects were chosen to minimize variability. An observer blind to experimental conditions performed the entire analysis.

**Figure 5:**

For each vole, levels of relevant protein from entire mPFC (Cg and PLC), NAcc and PVN were measured only once with Western blotting. Quantification was performed only once using ImageJ software.

**Figure 6:**

Voles were allowed 3 d of post-operative recovery. All behavioral responses for each animal from groups with different treatments were scored and quantified once. Data were excluded if tips were located in other brain regions.

**Figure 7 and 8:**

Optogenetic behavioral tests started 6 weeks after virus injection. All behavioral responses for each animal from every group were scored and quantified once.To detect the c-fos expression actived by optogenetic, voles were placed in the open field for 5 min and were sacrificed 90 min later. For each vole, six brain sections were counted for quantifying c-fos positive cells in the PrL (bule light: right; yellow light: right and left).

**Statistical reporting**

- Statistical analysis methods should be described and justified
- Raw data should be presented in figures whenever informative to do so (typically when N per group is less than 10)
- For each experiment, you should identify the statistical tests used, exact values of N, definitions of center, methods of multiple test correction, and dispersion and precision measures (e.g., mean, median, SD, SEM, confidence intervals; and, for the major substantive results, a measure of effect size (e.g., Pearson's r, Cohen's d)
- Report exact p-values wherever possible alongside the summary statistics and 95% confidence intervals. These should be reported for all key questions and not only when the p-value is less than 0.05.

Please outline where this information can be found within the submission (e.g., sections or figure legends), or explain why this information doesn’t apply to your submission:

We have uploaded a relevant additional source data file.

**In the last paragraph of materials and method, statistical analysis methods had been described in detail.**

All raw data have been presented in source data files.

**Other information can be found in the figure legend.**

**Fig 1.** Independent sample t-tests (two-tailed) were used to assess differences in behaviors in the OFT. PC vs. PD (male: p<0.01; female: p<0.05). The values are expressed as means + SEM, error bars indicate SEM.

Independent sample t-tests (two-tailed) were used to assess percentage of time in light between PC and PD groups. PC vs. PD (male: p<0.05; female: p<0.01). The values are expressed as means + SEM, error bars indicate SEM.

The data from SPT were analyzed using two-way ANOVA (factors: treatment × stimulus), paired t-tests were used to compare approach/avoidance with Bonferroni correction for multiple comparisons (given that two groups were examined in Experiment 1, the threshold for signiﬁcance was set as p<0.025). object stimulus vs. social stimulus (PC male: p<0.025, PD male: p = 0.053; PC female: p<0.025, PD female: p = 0.141); PC vs. PD (social stimulus male: p<0.01). The values are expressed as means + SEM, error bars indicate SEM.

**Fig 2.** The two-way ANOVA (factors: treatment × stimulus) was used to compare the number of OT/ c-fos double positive-labeling neurons in the PVN after exposure to a juvenile vole compared to a toy. Toy vs. Vole (PC male: p<0.01; PC female: p<0.01); PC vs. PD (female after exposure to a juvenile vole: p<0.01). The values are expressed as means + SEM, error bars indicate SEM.

**Fig 3.** Neuronal activities in the PrL during the SAT were analyzed by two-way ANOVA (factors: treatment × stimulus). Toy vs. Vole (PC male: p<0.01; PC female: p<0.01); PC vs. PD (female or male after exposure to a juvenile vole: p<0.01). The values are expressed as means + SEM, error bars indicate SEM.

Neuronal activities in the NAc during the SAT were analyzed by two-way ANOVA (factors: treatment × stimulus). Toy vs. Vole (PC female: p<0.01); PC vs. PD (Male after exposure to a juvenile vole: p< 0.01). The values are expressed as means + SEM, error bars indicate SEM.

**Fig 4.** In the PVN, the number of OT positive neurons were analyzed using two-way ANOVA (factors: treatment × sex). PC vs. PD (male: p<0.01; female: p<0.01); male vs. female (PC: p<0.01). The values are expressed as means + SEM, error bars indicate SEM.

**Fig 5.** Independent sample t-tests were used to assess levels of OTR, V1aR and AVP protein in different brain regions. In the mPFC, PC vs. PD (male OTR: p<0.01, female OTR: p<0.01; male V1aR: p<0.05, female V1aR: p = 0. 594; male AVP: p = 0.253, female AVP: p = 0.408); In the NAc, PC vs. PD (male OTR: p = 0.563, female OTR: p = 0.665; male V1aR: p = 0.092, female V1aR: p = 0.663; male AVP: p = 0.151, female AVP: p = 0.437); In the PVN, PC vs. PD (male OTR: p = 0.616, female OTR: p = 0.273; male V1aR: p = 0.179, female V1aR: p = 0.830; male AVP: p = 0.340, female AVP: p = 0.687).

The values are expressed as means + SEM, error bars indicate SEM.

**Fig 6.** The data from OFT (Experiment 5) were analyzed using one-way ANOVA followed by SNK post-hoc tests (Male: 10ng OT vs. CSF (p<0.05), 10ng OT vs. 1ng OT (p<0.05), 10ng OT vs. 10ng OT/100ng OTA (p<0.05), 10ng OT vs. 10ng OT/10ng V1aRA (p<0.05) ; 10ng OT/10ng OTA vs. CSF (p<0.05), 10ng OT/10ng OTA vs. 1ng OT (p<0.05), 10ng OT/10ng OTA vs. 10ng OT/100ng OTA, 10ng OT/10ng OTA vs. 10ng OT/10ng V1aRA (p<0.05). Female: 1ng OT vs. CSF (p<0.05), 1ng OT vs. 1ng OT/10ng OTA (p<0.05), 1ng OT vs. 1ng OT/100ng OTA (p<0.05); 10ng OT vs. CSF (p<0.05), 10ng OT vs. 1ng OT/10ng OTA (p<0.05), 10ng OT vs. 1ng OT/100ng OTA (p<0.05); 10ng OT/10ng V1aRA vs. CSF (p<0.05), 10ng OT/10ng V1aRA vs. 1ng OT/10ng OTA (p<0.05), 10ng OT/10ng V1aRA vs. 1ng OT/100ng OTA (p<0.05)). Bars without the same letters are signiﬁcantly different. The values are expressed as means + SEM, error bars indicate SEM.

The data from the SPT were analyzed using two-way ANOVA (factors: treatment × stimulus), paired t-tests were used to compare approach/avoidance with Bonferroni correction for multiple comparisons (given that five groups were examined in Experiment 5, the threshold for signiﬁcance was set p<0.0083). object stimulus vs. social stimulus (10ng OT male: p<0.0083, 10ng OT/10ng OTA male: p < 0.008; 1ng OT female: p<0.0083, 10ng OT female: p<0.0083, 10ng OT/10ng V1aRA female: p<0.0083).The values are expressed as means + SEM, error bars indicate SEM.

**Fig 7.** Independent sample t-tests (two-tailed) were used to assess differences in behaviors in the OFT. Ctrl vs. ChR2 (male: p<0.01; female: p<0.01). The values are expressed as means ± SEM, error bars indicate SEM.

Independent sample t-tests (two-tailed) were used to assess the number of c-fos positive neurons between Ctrl and ChR2 groups. Ctrl vs. ChR2 (male: p<0.01; female: p<0.01). The values are expressed as means + SEM, error bars indicate SEM.

The data from SPT were analyzed using two-way ANOVA (factors: treatment × stimulus), paired t-tests were used to compare approach/avoidance with Bonferroni correction for multiple comparisons (given that two groups were examined in Experiment 6, the threshold for signiﬁcance was set as p<0.025). object stimulus vs. social stimulus (Ctrl male: p = 0.113, ChR2 male: p <0.025; Ctrl female: p = 0.668, ChR2 female: p<0.025); Ctrl vs. ChR2 (social stimulus female: p < 0.025). The values are expressed as means + SEM, error bars indicate SEM.

**Fig 8.** Independent sample t-tests (two-tailed) were used to assess differences in behaviors in the OFT. Ctrl vs. eNpHR3.0 (male: p<0.01; female: p<0.01). The values are expressed as means ± SEM, error bars indicate SEM.

Independent sample t-tests (two-tailed) were used to assess the number of c-fos positive neurons between Ctrl and eNpHR3.0 groups. Ctrl vs. eNpHR3.0 (male: p<0.01; female: p<0.01). The values are expressed as means + SEM, error bars indicate SEM.

The data from SPT were analyzed using two-way ANOVA (factors: treatment × stimulus), paired t-tests were used to compare approach/avoidance with Bonferroni correction for multiple comparisons (given that two groups were examined in Experiment 6, the threshold for signiﬁcance was set as p<0.025). object stimulus vs. social stimulus (Ctrl male: p<0.025, eNpHR3.0 male: p = 0.154; Ctrl female: p<0.025, eNpHR3.0 female: p = 0.642). The values are expressed as means + SEM, error bars indicate SEM.

(For large datasets, or papers with a very large number of statistical tests, you may upload a single table file with tests, Ns, etc., with reference to sections in the manuscript.)

**Group allocation**

- Indicate how samples were allocated into experimental groups (in the case of clinical studies, please specify allocation to treatment method); if randomization was used, please also state if restricted randomization was applied
- Indicate if masking was used during group allocation, data collection and/or data analysis

Please outline where this information can be found within the submission (e.g., sections or figure legends), or explain why this information doesn’t apply to your submission:

**This information can be found in results.**

**Figure 1:**

At 70 days of age, subjects were randomly divided into the control group (PC group: 7 females and 7 males) and PD group (7 females and 7 males).

**Figure 2, 3 and 4:**

To detect the c-fos or c-fos/OT double-labeled neurons activated by exposure to a juvenile vole or a toy, experimental mandarin voles from the PC (n = 5 males, 5 females) and PD (n = 5 males, 5 females) groups were anesthetized with pentobarbital sodium after 90 min.

To investigate whether PD affect the number of OT-IR neurons in the PVN, three hours after the social preference test, mandarin voles were anesthetized with pentobarbital sodium in PC (n = 4 males, 4 females) and PD (n = 4 males, 4 females) groups.

Brains were removed from the skull and placed in 4% paraformaldehyde three days. Brains were then dehydrated in 30% sucrose solution until saturated at 4°C for immunohistochemistry.

**Figure 5:**

Voles from the PC (n = 6 males, 6 females) and PD (n = 6 males, 6 females) groups were sacrificed by rapid decapitation at 70 days of age. All brains were harvested, frozen on dry ice, and stored at -80°C until Western blotting.

**Figure 6:**

At 70 days of age, mandarin voles in the PD group (72 voles including thirty-six males and thirty-six females) were randomly allocated into experimental groups after stereotaxic cannulation (Male: CSF: n = 6, 1ng OT: n = 6, 10ng OT: n = 6, 10ng OT /10 ng OTA: n = 6, 10ng OT /100 ng OTA: n = 6, 10ng OT/10ng V1aRA: n = 6; Female: CSF: n = 6, 1ng OT: n = 6, 10ng OT: n = 6, 1ng OT /10 ng OTA: n = 6, 1ng OT /100 ng OTA: n = 6, 10ng OT/10ng V1aRA: n = 6). Animals with missed cannula placements in other brain regions were excluded from analyses. The final size of each group was 6 except for the 10 ng OT/100 ng OTA male group which numbered 5, and the 1 ng OT/10 ng OTA and 1 ng OT/100 ng OTA female groups (both n = 5). Male: CSF: n = 6, 1ng OT: n = 6, 10ng OT: n = 6, 10ng OT /10 ng OTA: n = 6, 10ng OT /100 ng OTA: n = 5, 10ng OT/10ng V1aRA: n = 6 ; Female: CSF: n = 6, 1ng OT: n = 6, 10ng OT: n = 6, 1ng OT /10 ng OTA: n = 5, 1ng OT /100 ng OTA: n = 5, 10ng OT/10ng V1aRA: n = 6.

**This information can be found in results.**

**Figure 7:**

Quantification of c-fos positive neurons in the PrL of males (Ctrl: n = 4; ChR2: n = 5) and females (Ctrl: n = 4; ChR2: n = 5) after photostimulation. Optogenetic activation of oxytocinergic fibers in the PrL increased social preference of males (Ctrl: n = 6; ChR2: n = 6) and females (Ctrl: n = 6; ChR2: n = 6). Activation of PVN-PrL oxytocinergic projection significantly increased the percentage of time in the central area of males (Ctrl: n = 6; ChR2: n = 6) and females (Ctrl: n = 6; ChR2: n = 6), but did not influence total distance in males (Ctrl: n = 6; ChR2: n = 6) and females (Ctrl: n = 6; ChR2: n = 6).

**Figure 8:**

Quantification of c-fos positive neurons in the PrL of males (Ctrl: n = 4; eNpHR3.0: n = 4) and females (Ctrl: n = 4; eNpHR3.0: n = 4) after yellow light photostimulation. Optogenetic inhibition of oxytocinergic fibers in the PrL increased social preference of males (Ctrl: n = 6; eNpHR3.0: n = 6) and females (Ctrl: n = 6; eNpHR3.0: n = 6). Inhibition of PVN-PrL oxytocinergic projection significantly decreased the percentage of time in the central area of males (Ctrl: n = 6; eNpHR3.0: n = 6) and females (Ctrl: n = 6; eNpHR3.0: n = 6), but does not influence total distance in males (Ctrl: n = 6; eNpHR3.0: n = 6) and females (Ctrl: n = 6; eNpHR3.0: n = 6).

**Additional data files (“source data”)**

- We encourage you to upload relevant additional data files, such as numerical data that are represented as a graph in a figure, or as a summary table
- Where provided, these should be in the most useful format, and they can be uploaded as “Source data” files linked to a main figure or table
- Include model definition files including the full list of parameters used
- Include code used for data analysis (e.g., R, MatLab)
- Avoid stating that data files are “available upon request”

Please indicate the figures or tables for which source data files have been provided:

We have uploaded a relevant additional source data file.

**Figure 1- Source Data 1**

**Figure 2- Source Data 2**

**Figure 3- Source Data 3**

**Figure 4- Source Data 4**

**Figure 5- Source Data 5**

**Figure 6- Source Data 6**

**Figure 7- Source Data 7**

**Figure 8- Source Data 8**
